# Altered Left Ventricular Ion Channel Transcriptome in a High-Fat-Fed Rat Model of Obesity: Insight into Obesity-Induced Arrhythmogenesis

**DOI:** 10.1155/2016/7127898

**Published:** 2016-09-25

**Authors:** Reza Ashrafi, Marianne Yon, Lucy Pickavance, Joseph Yanni Gerges, Gershan Davis, John Wilding, Kun Jian, Henggui Zhang, George Hart, Mark Boyett

**Affiliations:** ^1^Department of Obesity & Endocrinology, Institute of Ageing and Chronic Disease, Faculty of Health & Life Sciences, University of Liverpool, 4th Floor, UCD, Duncan Building, Daulby Street, Liverpool L69 3GA, UK; ^2^Institute of Cardiovascular Sciences, University of Manchester, Core Technology Facility, 46 Grafton Street, Manchester M13 9NT, UK; ^3^Biological Physics Group, School of Physics & Astronomy, University of Manchester, Schuster Building, Oxford Road, Manchester M13 9PL, UK

## Abstract

*Introduction*. Obesity is increasingly common and is associated with an increased prevalence of cardiac arrhythmias. The aim of this study was to see whether in obesity there is proarrhythmic gene expression of ventricular ion channels and related molecules.* Methods and Results*. Rats were fed on a high-fat diet and compared to control rats on a normal diet (*n* = 8). After 8 weeks, rats on the high-fat diet showed significantly greater weight gain and higher adiposity. Left ventricle samples were removed at 8 weeks and mRNA expression of ion channels and other molecules was measured using qPCR. Obese rats had significant upregulation of Ca_v_1.2, HCN4, K_ir_2.1, RYR2, NCX1, SERCA2a, and RYR2 mRNA and downregulation of ERG mRNA. In the case of HCN4, it was confirmed that there was a significant increase in protein expression. The potential effects of the mRNA changes on the ventricular action potential and intracellular Ca^2+^ transient were predicted using computer modelling. Modelling predicted prolongation of the ventricular action potential and an increase in the intracellular Ca^2+^ transient, both of which would be expected to be arrhythmogenic.* Conclusion*. High-fat diet causing obesity results in arrhythmogenic cardiac gene expression of ion channels and related molecules.

## 1. Introduction

Obesity is an important developing health issue worldwide and the percentage of people classified as obese (BMI > 30 kg/m^2^) has more than doubled in the last half century [1], which has significant potential consequences for the cardiovascular health of the population. The process by which individuals become obese is a multifactorial one with consumption of a diet with an excessive energy content arising mainly from high levels of refined carbohydrates and saturated fats, combined with a reduced level of physical activity, a common observation in epidemiological studies [2]. While, in a heterogeneous population, obese individuals will vary in whether their diet contains a higher proportion of saturated fats or refined carbohydrates, there are many studies reporting that diets rich in saturated fats lead to obesity [3].

Obesity is linked to an increased likelihood of atrial [4] and ventricular [5] arrhythmias and sudden cardiac death [6]. The mechanisms responsible for the increase in arrhythmias are not understood. In obese individuals who succumbed to sudden cardiac death, an increase in ventricular ectopy has been observed, which in many cardiac disease states is linked to prolonged ventricular arrhythmias and death [7]. An increase in the corrected QT interval in obese individuals has been observed [8] and an increase in the QT interval is a common finding in many arrhythmogenic disorders. The increase in the QT interval has been suggested to be the result of high levels of circulating fatty acids interfering with ventricular repolarization [9]. However, the increase in the QT interval could also be the result of changes in ventricular ion channel expression. For example, in heart failure, there are changes in ventricular ion channel expression and an increase in the QT interval and ventricular arrhythmias [10].

The rationale behind this study is as follows: in light of the rapidly increasing number of obese patients in many countries, there is a lack of data on the potential mechanisms for the higher rates of arrhythmias seen in obese patients when compared to other conditions associated with arrhythmias such as channelopathies where mechanisms are well researched and understood. The objective of this study as part of a wider study into the effects of dietary obesity was to gain an insight into some of the potential mechanisms underlying obesity-induced arrhythmogenesis by investigating the expression of a variety of key cardiac ion channels (and related molecules) in a rat model of obesity based on high saturated fat consumption.

## 2. Methods

### 2.1. Animals and Diets

Age-matched male Wistar rats (~250 g; Charles River, Margate, UK) were arbitrarily assigned into two groups and fed with either a high-fat diet (HFD) or control diet for eight weeks (*n* = 8/group). These provided either 40% of calories (high-fat diet) or 10% of calories (control diet) from saturated fatty acids (sourced mainly from lard) or polyunsaturated fatty acids (sourced mainly from soybean oil), respectively. The rest of the diet was made up of 20% protein in both groups and the remaining calories were carbohydrates mainly from corn starch (Research Diets, Inc., New Brunswick, NJ, USA). Both diets contained equal amounts of the antioxidant tert-butylhydroquinone (tBHQ) to preserve the component fats. Comparative diet compositions per feed batch are shown in Table 1.

All animals were singly housed, maintained on a standard 12-hour on/off light cycle, and provided with food and water* ad libitum*. Body weight was measured weekly and food intake daily. After eight weeks, all animals were killed with a rising concentration of carbon dioxide, followed by cervical dislocation. A single epididymal fat pad was then dissected from each rat and weighed. As a measure of adiposity, this mass was later expressed relative to the final body weight, as previously described [11]. Hearts were dissected from all rats and placed in physiological Hartmann's solution, where standard anatomical markers were used as reference points before removing a 5 mm strip of the midleft ventricular free wall, which was snap-frozen in liquid N_2_ (−80°C). All experiments were undertaken in accordance with the UK Animals (Scientific Procedures) Act 1986.

### 2.2. RNA Isolation and Quantitative Gene Expression Profiling

Frozen tissue was subsequently cut into 20 *μ*m sections on a cryostat before RNA was isolated using Qiagen RNeasy minicolumns (Qiagen, Crawley, UK). RNA quality was assessed using commercially available spectrophotometry (Nanodrop, Thermo Scientific, Loughborough, UK) and an equal amount of RNA from each sample was then amplified to cDNA using high-capacity RNA to cDNA for quantitative PCR (Applied Biosystems, Warrington, UK). Quantitative PCR was performed using custom preloaded low-density TaqMan array microfluidic cards, Universal Mastermix II, and a 7900HT fast real-time PCR system (Applied Biosystems). Relative expression of the gene targets was made using the “ΔCt method,” in which the abundance of target genes is normalised to the abundance of a housekeeper (or reference) gene, 18-s in this study. 18-s was chosen as the housekeeper after an analysis of several potential housekeeper genes (18-s, GAPDH, and Cx43) as it had the smallest *M* value, as assessed by geNORM analysis [12] (StatMiner 4.1, Integromics, Uckfield, UK).

Relative abundance of mRNA is presented in arbitrary units referenced to 18-s expression for all gene targets.

### 2.3. Immunofluorescence

In the case of HCN4, protein expression was measured using immunofluorescence with quantitative signal intensity as described previously [13, 14]. Frozen sections of the midleft ventricular free wall that previously had been isolated (*n* = 8/group) were placed on a cryostat and 10 *μ*m thick sections sectioned and placed on a Poly-Prep slide (Sigma-Aldrich, Dorset, UK). Sections were fixed in buffered 10% formalin solution and sections were then washed in 1x phosphate buffered saline (PBS) before permeabilisation with 0.1% Triton-X 100 before nonspecific blocking with bovine serum albumin (BSA) diluted in PBS. Sections were then incubated overnight at 4°C in the dark with 1 : 100 primary antibody (rabbit anti-HCN4, Alomone, Jerusalem, Israel) in 1% BSA in PBS before washing with PBS and incubation in the dark at room temperature with 1 : 500 secondary antibody (donkey anti-rabbit IgG, rhodamine, Millipore, Watford, UK) in 1% BSA in PBS for 2 h. A negative control was undertaken using a section from a control animal incubated with secondary antibody but not primary antibody: no labelling was observed (in keeping with other studies from our group [15, 16]). Sections were then washed with PBS before mounting with Vectashield (Vectorlabs, Peterborough, UK). Confocal microscopy (Zeiss LSM5 PASCAL, Zeiss, Cambridge, UK) was used for image acquisition before quantitative immunofluorescence signal intensity measurements were carried out using Volocity software (PerkinElmer, Beaconsfield, UK). For each sample, 5 regions of the left ventricular cryostat sample were analysed with an identical field of view size and the signal intensity value was recorded before being averaged.

### 2.4. Mathematical Modelling of the Action Potential

Before specific modelling of the funny current (*I*_*f*_), the original model in Pandit et al. [17] was modified to incorporate experimental data from Cerbai et al. [18] more specifically. Further details are available in the supplementary data. The activation curve from Cerbai et al. [18] was fitted using the Boltzmann distribution (*V*_*H*_ = −87.74 mV, *k* = −10.12):

(1) $$\begin{array}{c} y\infty =\frac{1.0}{1.0+e^{\left(V+87.74\right)/10.12}}, \end{array}$$

where *y∞* is the steady-state value of the activation variable, *y*, and *V* is the membrane potential. The time constant of *I*_*f*_ activation was reformulated based on data from Cerbai et al. [18]:

(2) $$\begin{array}{c} \tau_{y}=\frac{1.0}{0.1177*e^{\left(V+86.78\right)/29.5}}+08141*e^{-\left(V+86.78\right)/14.75}, \end{array}$$

where *τ*_*y*_ is the time constant of *y*. The rate of change in the activation variable, *y*, was calculated from

(3) $$\begin{array}{c} \frac{dy}{dt}=\frac{y\infty -y}{\tau_{y}}. \end{array}$$

Finally, *I*_*f*_ was calculated assuming that it is carried by a mixture of Na^+^ and K^+^:

(4) $$\begin{array}{c} I_{f}=g_{f}y\left[\;f_{Na}\left(\;V-E_{Na}\;\right)+f_{K}\left(\;V-E_{K}\;\right)\;\right], \end{array}$$

where *g*_*f*_ is the maximum conductance, *f*_Na_ and *f*_K_ are the fractions of *I*_*f*_ carried by Na^+^and K^+^ (*f*_Na_ = 0.2; *f*_K_ = 1 − *f*_Na_), and *E*_Na_ and *E*_K_ are the equilibrium potentials of Na^+^and K^+^. *g*_*f*_ (0.0043 *μ*S) was obtained by matching simulated current traces of *I*_*f*_ to experimental data.

In normal and obesity conditions, the models were run for a 5 s period to obtain a stable state condition before a sequence of external stimulus pulses (with an amplitude of 0.8 nA, duration of 6 ms, and frequency of 1 Hz) were applied to evoke an action potential. In order to evaluate the relative role of each of the remodelled ionic currents, simulations were also performed by considering changes to each individual ionic current alone.

To simulate the effects of obesity, the channel conductance for each of the presumed remodelled ionic currents in Pandit et al.'s model [17] was scaled according to the measured average percentage change of the corresponding mRNA between the control and high-fat diet groups. Using this mRNA change, the original baseline equation variables were altered for the high-fat diet group before the model was run to produce an action potential as described previously [19, 20]. No changes were made to ion concentrations in the calculations.

A summary of the relative expression differences in the high-fat group used to produce the modelled action potentials is shown in Table 2.

### 2.5. Statistical Analysis

Grouped mean data are reported as mean ± SEM. Between-group comparisons were made using Student's *t*-test if a Shapiro-Wilk test of normality was passed or a rank sum test if the data was not normally distributed. Results were considered significant when *P* < 0.05.

## 3. Results

### 3.1. Dietary Outcomes

Over the course of the study, high-fat-diet-fed rats consumed more energy (28.1 ± 1.0 × 10^3^ versus 23.7 ± 0.9 × 10^3^ kJ; +18%, *P* = 0.02 versus controls) and gained more weight (232.9 ± 12.4 versus 308.6 ± 18.5; +32%; *P* < 0.01) than did control animals. The latter was consistent with greater accrual of adipose tissue in high-fat-diet-fed rats (+1.449% ± 0.010 versus 1.032 ± 0.08; +40%; *P* < 0.01). Due to the high energy content of the high-fat diet, lesser intake was required to induce weight gain in this group compared to controls (−10%; *P* = 0.02).

### 3.2. Transcriptome of Major Ion Channels Active during the Action Potential

**Figure 1:**
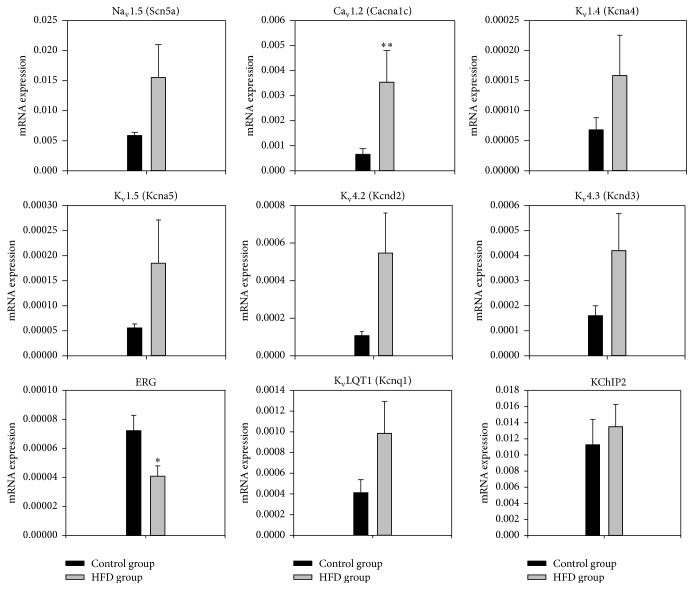
Normalised to 18-s expression of mRNA for major ion channels active during the action potential in the left ventricle of the control and obesity groups in arbitrary units referenced to the housekeeper gene 18-s. Means ± SEM shown (*n* = 8/group). ^*∗*^Significantly different from the control group (*P* < 0.05). ^*∗∗*^Significantly different from the control group (*P* < 0.01).

Expression of ion channels in the left ventricle of the obese rats (*n* = 8) was measured at the mRNA level using quantitative PCR and compared to that in the control lean rats (*n* = 8). Expression of the principal Na^+^ channel, Na_v_1.5 (Scn5a), responsible for the Na^+^ current (*I*_Na_) tended to be greater in the obese group, but not significantly so (Figure 1). However, expression of the principle L-type Ca^2+^ channel, Ca_v_1.2 (Cacna1c), responsible for the L-type Ca^2+^ current (*I*_Ca,L_) was significantly increased in the obese group with an increase greater than 500% (*P* < 0.01). Expression of K_v_1.4 (Kcna4), K_v_4.2 (Kcnd2), K_v_4.3 (Kcnd3), and KChIP2 (Kcnip2), responsible for the transient outward K^+^ current (*I*_to_), K_v_1.5 (Kcna5), responsible for the ultrarapid delayed rectifier K^+^ current (*I*_K,ur_) in humans but the steady-state current (*I*_K,ss_) in rats, and K_v_LQT1 (Kcnq1), responsible for the slow delayed rectifier K^+^ current (*I*_K,s_), all tended to be greater in the obese group, but not significantly so. In contrast, expression of ERG (K_v_11.1), responsible for the rapid delayed rectifier K^+^ current (*I*_K,*r*_), was significantly decreased in the obese group (*P* = 0.029, as shown in Figure 1).

### 3.3. Transcriptome of Major Ion Channels Active during Diastole

**Figure 2 fig2:**
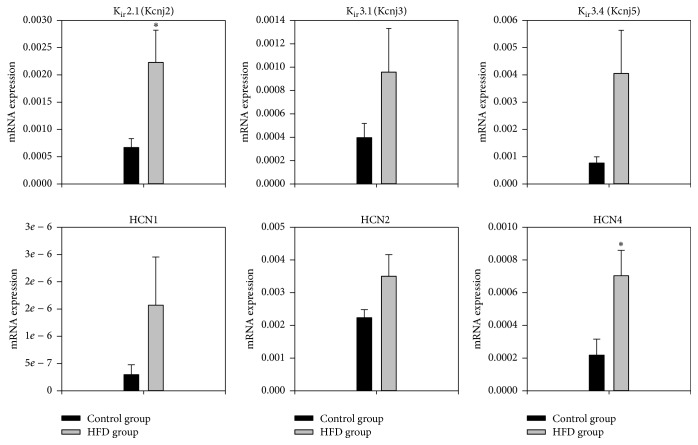
Normalised to 18-s expression of mRNA for major ion channels active largely during diastole in the left ventricle of the control and obesity groups in arbitrary units referenced to the housekeeper gene 18-s. Means ± SEM shown (*n* = 8/group). ^*∗*^Significantly different from the control group (*P* < 0.05).

Three channel isoforms, HCN1, HCN2, and HCN4, are responsible for the funny current (*I*_*f*_), an important pacemaker current. Expression of all three isoforms tended to be greater in the obese group but the increase in HCN4 (322%) was significant (*P* = 0.03). In the obese group, there was significantly increased expression of K_ir_2.1 (Kcnj2) responsible for the background inward rectifier K^+^ current (*I*_K,1_, Figure 2). Expression of K_ir_3.1 (Kcnj3) and K_ir_3.4 (Kcnj5) responsible for the ACh-activated K^+^ current (*I*_K,ACh_) tended to be greater in the obese group, but not significantly so (Figure 2). There was a nonsignificant increase in the expression of the Na^+^-K^+^ATPases 1–3.

### 3.4. Transcriptome of Intracellular Ca^2+^-Handling Molecules

**Figure 3:**
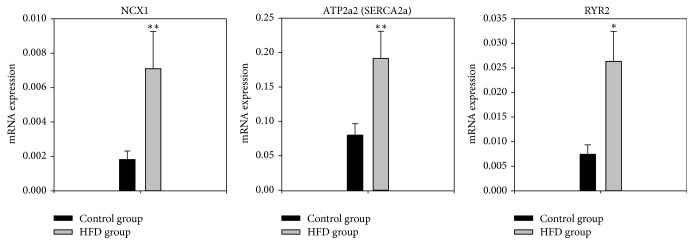
Normalised to 18-s expression of mRNA for major Ca^2+^-handling molecules in the left ventricle of the control and obesity groups in arbitrary units referenced to the housekeeper gene 18-s. Means ± SEM shown (*n* = 8/group). ^*∗*^Significantly different from the control group (*P* < 0.05). ^*∗∗*^Significantly different from the control group (*P* < 0.01).

Intracellular Ca^2+^ plays an important role in arrhythmogenesis and three important intracellular Ca^2+^-handling molecules were investigated: NCX1 (the Na^+^-Ca^2+^exchanger), SERCA2a (ATP2a2, the sarcoplasmic reticulum (SR) Ca^2+^ pump), and RYR2 (the ryanodine receptor, the SR Ca^2+^ release channel). Expression of all three was significantly increased in the obese group (Figure 3) by over 200% for NCX1 and RYR2 (*P* < 0.01).

### 3.5. Immunolabelling of HCN4

**Figure 4:**
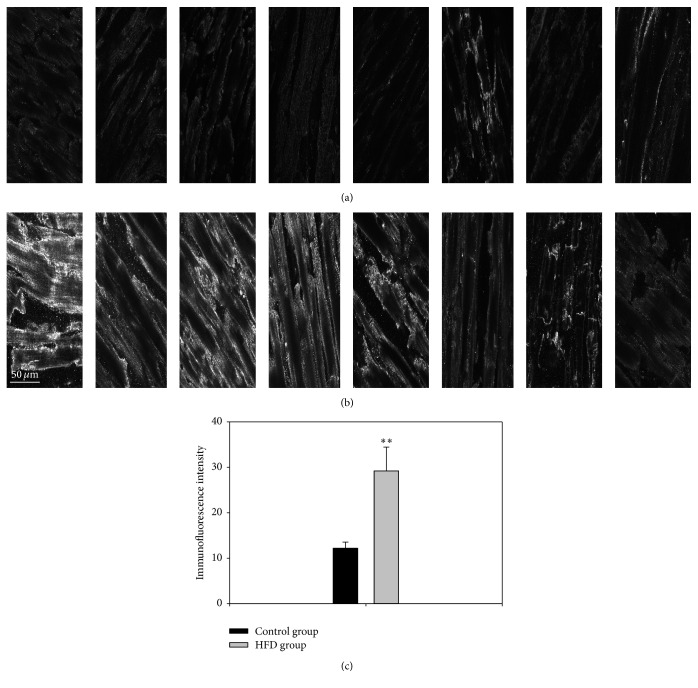
Representative images of HCN4 immunolabelling (white signal) in the left ventricle of control (a) and obese (b) animals. (c) Mean ± SEM signal intensity in arbitrary units of HCN4 immunolabelling in the left ventricle of the control and obesity groups (*n* = 8/group). ^*∗∗*^Significantly different from the control group (*P* < 0.01).

In the case of the pacemaker ion channel, HCN4, immunohistochemistry was used to show whether the mRNA changes led to corresponding changes at the protein expression level. Signal intensity measurements of HCN4 protein expression support the notion that increased mRNA expression was associated with higher protein expression (*P* < 0.01) (Figure 4).

### 3.6. Potential Effect of Obesity on the Ventricular Action Potential as Predicted by Action Potential Modelling

**Figure 5:**
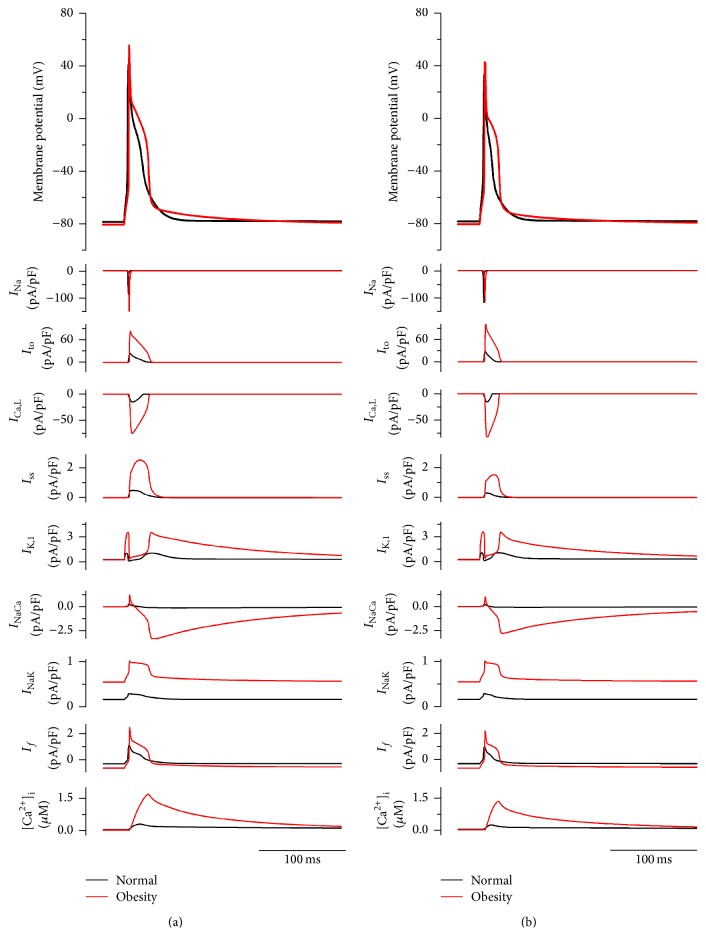
Simulated rat ventricular endocardial ((a) ENDO) and epicardial ((b) EPI) action potentials and underlying ionic currents and intracellular Ca^2+^ concentration in control and obesity conditions.

Figure 5 shows simulated action potentials (top row) of rat endocardial (Figure 5(a)) and epicardial (Figure 5(b)) ventricular cells in control and obesity conditions, accompanied by underlying ionic currents and the intracellular Ca^2+^ concentration. In both cell models, remodelled ion channels in the obesity condition produced increased amplitude of the action potential, elevation in the plateau phase, and an increase in the action potential duration (Figure 5). Notably, there was a pronounced slow tail of repolarization following the action potential; slow tails of repolarization following the rat ventricular action potential have been reported in experiments [21]. In the obesity condition, simulation results also showed that the amplitude of the intracellular Ca^2+^ concentration was increased (Figure 5).

### 3.7. Effects of Each of the Remodelled Ion Channels on the Ventricular Action Potential

**Figure 6:**
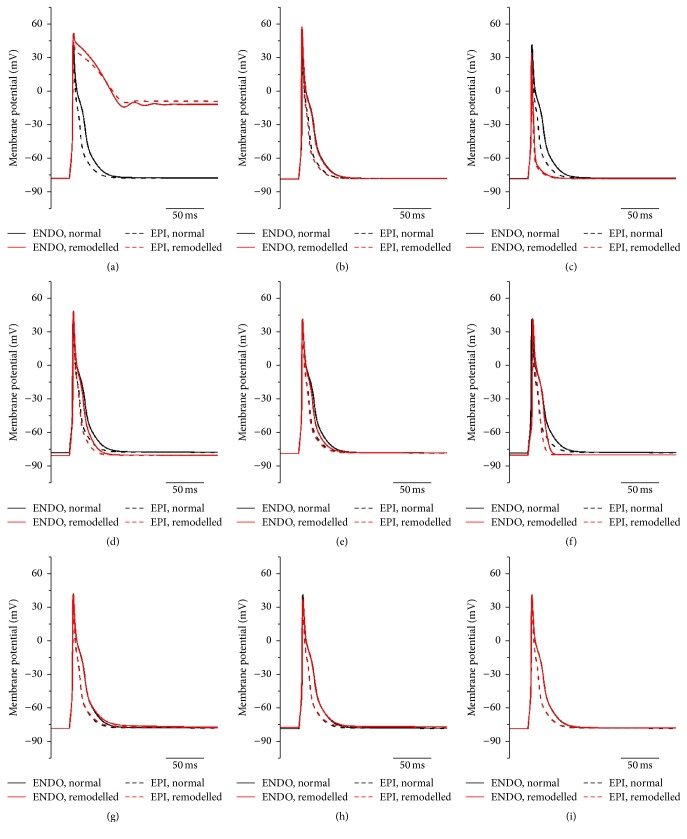
Simulated rat ventricular endocardial and epicardial action potentials in control and obesity conditions when each of the remodelled ion channels/ionic currents was modified one at a time. (a) Only *I*_Ca,L_ considered. (b) Only *I*_Na_ considered. (c) Only *I*_to_ considered. (d) Only *I*_NaK_ considered. (e) Only *I*_ss_ considered. (f) Only *I*_K,1_ considered. (g) Only *I*_NaCa_ considered. (h) Only *I*_*f*_ considered. (i) Only SR Ca^2+^ uptake and release considered (*J*_Up_ and *J*_Rel_, resp.).

Potential effects of each of the remodelled ion channels on the ventricular action potential were investigated. Results are shown in Figure 6. The potential increase in *I*_Ca,L_ alone produced a dramatic increase in the duration of the plateau phase, leading to a failure of repolarization; consequentially, the models failed to produce a full action potential (Figure 6(a)). The potential increase in *I*_to_ alone abbreviated the action potential and reduced the action potential amplitude (Figure 6(c)). The potential increases in *I*_K,ss_ (Figure 6(e)) and *I*_K,1_ (Figure 6(f)) resulted in small abbreviations of the action potential. Upregulation of *I*_NaCa_ (Figure 6(g)) resulted in a small delay in phase 3 repolarization.

Therefore, the simulations suggest that the obesity-induced changes in ion channels could result in prolongation of action potential primarily as a result of an increase in *I*_Ca,L_, the effects of which are offset to a degree by increases in *I*_to_ and *I*_K,1_.

## 4. Discussion

We have shown that a diet rich in saturated fats leading to obesity results in significant upregulation of Ca_v_1.2, HCN4, K_ir_2.1, NCX1, SERCA2a, and RYR2 mRNA and significant downregulation of ERG mRNA in the left ventricle. mRNA changes when modelled caused significant abnormalities in the modelled action potential. Such changes may help define the substrate underlying obesity-induced ventricular arrhythmias.

### 4.1. Channels and Exchanger Proteins

The upregulation of Ca_v_1.2 mRNA in obesity (Figure 1), if translated into an increase in *I*_Ca,L_, is expected to be proarrhythmic. Action potential modelling (Figure 6(a)) showed that an increase in Ca_v_1.2 and *I*_Ca,L_ will lead to prolongation of phase 2 of the action potential and the QT interval. It is also expected to directly and indirectly promote the formation of early and delayed afterdepolarizations (EADs and DADs), which can generate ectopic beats and arrhythmias [22]. The action potential modelling showed a large increase in Na^+^-Ca^2+^ exchange current, *I*_NaCa_ (Figure 5). Figure 6(g) suggests that the effect of an increase in *I*_NaCa_ simply as a result of the upregulation of NCX1 mRNA is small; however, Figure 6 shows that the large Ca^2+^ transient helps to create a large *I*_NaCa_ current. In obesity, it is predicted that *I*_NaCa_ is a large inward current in diastole immediately after the action potential and it declines slowly as intracellular Ca^2+^ falls. The large inward *I*_NaCa_ generates a slow tail of repolarization after the action potential (Figure 6(g)). In cardiac hypertrophy and heart failure, upregulation of NCX1 has been repeatedly described [23]. There was upregulation of K_ir_2.1 mRNA (responsible for *I*_K,1_) in obesity (Figure 2). Overexpression of K_ir_2.1 and increase of *I*_K,1_ result in acceleration of the final phase of repolarization and a shorter action potential [24]. This is confirmed by the action potential modelling in Figure 6(f). An increase in *I*_K,1_ is also reported to cause an increase in the transmural dispersion of repolarization [25], facilitating reentry arrhythmias [26]. The upregulation of K_ir_2.1 (*I*_K,1_) may be compensatory to prevent incomplete repolarization and minimise prolongation of the action potential caused by the upregulation of Ca_v_1.2 (and *I*_Ca,L_, Figure 6(a)). There was downregulation of ERG mRNA (responsible for *I*_K,*r*_) in obesity (Figure 2). In the rat ventricle, ERG and *I*_K,*r*_ are not thought to be functionally important; however, in the human, reduced expression or drug blockade of ERG and *I*_K,*r*_ is a well-known cause of action potential/QT prolongation and ventricular arrhythmias [27].

We observed significant upregulation in HCN4 at the mRNA and protein levels (Figures 3 and 5) in the left ventricle in obesity, a key pacemaker channel. HCN channels are expressed in the working myocardium, but at a lower level than in the cardiac conduction system [28]. Upregulation of HCN4 has been observed in the ventricles of rats with hypertrophy resulting from pressure overload [29] and left ventricular hypertrophy is a common finding in obese individuals [30]. HCN4 protein levels in the sinus node and the total area of HCN4 expressing tissue within the sinus node have been shown to be increased in elderly obese rats [31]. In a postmyocardial infarction animal model, ventricular upregulation of HCN4 causes a high volume of ventricular ectopic beats and potentially prolonged ventricular arrhythmias [32]. In contrast, action potential modelling suggested that upregulation of HCN4 (and *I*_*f*_) has no effect on the rat ventricular action potential (Figure 6(h)).

In summary, the increase in the QT interval observed in clinically obese patients [8] may be due to increased *I*_Ca,L_ predominantly.

### 4.2. Ca^2+^-Handling Proteins

There was upregulation of SERCA2a mRNA (responsible for the SR Ca^2+^ pump) in obesity (Figure 3). Upregulation of mRNA for SERCA2a and the closely associated molecule, phospholamban, has previously been reported in obese rats [33]. The increased SERCA2a expression has previously been explained as a response to oxidative damage to the SR caused by excess free radical generation in obesity [34]. The upregulation of SERCA2a is expected to increase the level of Ca^2+^ in the SR and an increase in *I*_Ca,L_ in obesity is expected to have the same effect. Such an increase in the level of Ca^2+^ in the SR is expected to result in an increase in the intracellular Ca^2+^ transient and this is consistent with the predictions of the computer simulations (Figure 6). Several studies have suggested that increased SERCA2a activity leading to Ca^2+^ overload in the SR can lead to abnormal SR Ca^2+^ release and DADs leading to arrhythmias [35] although other work has shown increased SERCA2a to be antiarrhythmogenic [36]. In the early stages of left ventricular hypertrophy, SERCA2a has previously been shown to be upregulated [37]. There was upregulation of RYR2 mRNA (responsible for the SR Ca^2+^ release channel) in obesity (Figure 3). Together with the upregulation of Ca_v_1.2 and SERCA2a, this is expected to be the cause of the increase in the intracellular Ca^2+^ transient (Figure 6). Some of the respective increase observed in SERCA2a and NCX1 we have seen may be a response to the increase in RYR2 and the intracellular Ca^2+^ transient [38] and this may be compensatory to remove excess Ca^2+^ from the sarcoplasmic reticulum which can be caused by leak from RYR2 but further work would be needed to confirm this.

### 4.3. Overall Phenotype

The overall phenotype we had modelled here and as would be expected from the mRNA changes is to cause prolongation of the action potential with an increase in the plateau phase followed by a sharper phase 3 repolarisation slope. Clinically, this prolongation of the action potential would lead to QT prolongation which is very closely linked to clinical arrhythmias. Some of the changes we saw had conflicting effects on the AP with K_ir_2.1 (*I*_K,1_) in particular likely to be compensatory to some of the AP prolongation caused predominantly by Ca_v_1.2 and *I*_Ca,L_. Whilst we did not see a statistical difference in mRNA expression for the genes encoding the *I*_to_ current, the tendency for these to be increased would be a natural compensation to the increased Ca_v_1.2 and *I*_Ca,L_ and would try to abbreviate the increase in the plateau phase and action potential duration. Overall, while there were some gene changes that appear to oppose the proarrhythmic effect of others, the predominant modelled phenotype was arrhythmogenic with prolongation of the AP.

### 4.4. Limitations

This study has primarily been an exploratory study identifying areas of interest in what we believed to be an underresearched area but as with all studies there are limitations to the data presented here. The major limitation of this study is a lack of direct protein quantification to correlate with our mRNA results and then subsequently direct electrophysiological testing of the channels (assessed at an mRNA level here). Whilst the TaqMan system has been used with reproducible results by our group and others, the mRNA data we present cannot be said to be evidence of a protein level change or a physiological channel alteration. The computer modelling we used in this study has been used by other groups to assess initially the hypothetical effects of gene expression changes on cardiac electrophysiology and we have used it as a similar first step in assessment but it is acknowledged by our team that the computer modelling is an initial step with the results needing confirmation using direct electrophysiology testing. We hope that the results presented here could be taken forward and further research could be performed to assess these areas.

## 5. Conclusion

In dietary obesity, there are significant changes in the gene expression of ion channels in the left ventricle, which may predispose to arrhythmias. The changes may possibly reflect a specific genotype relating to obesity that may need differing treatments and clinical investigation compared to other groups. As the prevalence of obesity continues to increase, the observed genotype changes would be expected to correlate with a growing number of patients with clinical arrhythmias. Further studies are required to confirm the mRNA changes with protein and electrophysiological measurements and to elucidate the significance of these changes and the potential for targeted treatment in obese patients.

## Figures and Tables

**Table 1:** 

Ingredient	Control diet		High-fat diet	
	Grams	Kcal	Grams	Kcal
Casein 80 Mesh	200	800	200	800
L-Cystine	3	12	3	12
Corn starch	510	2040	208	820
Maltodextrin 10	90	360	90	360
Sucrose	100	400	100	400
Cellulose	50	0	50	0
Soybean oil	25	225	25	225
Lard	20	180	155.5	1400
tBHQ	0.038	0	0.031	0
Mineral mix	10	0	10	0
Dicalcium phosphate	13	0	13	0
Calcium carbonate	5.5	0	5.5	0
Potassium citrate	16.5	0	16.5	0
Vitamin mix	10	40	10	40
Chlorine bitartrate	2	0	2	0
Food dyes	0.05	0	0.05	0

Total	1055.9	4057	885.58	4057

**Table 2:** Simulated changes in current density based on the mRNA expression differences shown in Figures 1 [Fig fig2]–3.

Channel	Current	High fat
Na_v_1.5	*I*_Na_	+163.60%
Ca_v_1.2	*I*_Ca,L_	+441.28%
K_v_1.4, K_v_4.2, and K_v_4.3	*I*_to_	+240.45%
K_v_1.5	*I*_K,ss_	+232.73%
HCN4	*I*_*f*_	+222%
K_ir_2.1	*I*_K,1_	+233.83%
NCX1	*I*_NaCa_	+290.10%
SERCA2a	SR Ca^2+^ uptake	+139%
RYR2	SR Ca^2+^ release	+253.97%
ATP1*α*1-3	*I*_NaK_	251%
